# Anecdote of an Insane Clergyman before the American Revolution

**Published:** 1847-10

**Authors:** 


21. *Anecdote of an Insane Clergyman before the American Revolution.*—At one of the late Religious Anniversary Meetings in Boston, the *Rev. Dr. Pierce*, of Brookline, related an amusing anecdote of Samuel Coolridge, who graduated from Harvard University in 1724. At that time, it was customary, on the death of a king, or any of the royal family, for the clergy to preach a funeral sermon. Mr. Coolridge, who was a man of fine abilities, wrote a sermon, and became so deeply interested in the matter, as to become insane. His insanity was harmless however, and exhibited itself in a desire to go round and visit the clergy. On one occasion, he visited the Rev. Mr. Hedge, of Warwick. The Rev. gentleman invited him to attend church with him. On passing through a field, he noticed Mr. Coolridge collecting some green apples and placing them in his bosom. He made no remark about it, however. Mr. Coolridge took a seat just beneath the pulpit. After Mr. Hedge had well advanced in his sermon, Mr. Coolridge observed a man asleep. He took an apple and threw it at him, but did not hit him, and no disturbance was created. A few moments after, he observed another man asleep, he again threw an apple; this hit the man plumly on the head, and he aroused rather suddenly from his slumber. Mr. Hedge observing the manœuvre, and thinking to frown down all such conduct, looked sharply at Mr. Coolridge, but he, nothing abashed, looked up and said, "Go on with your business of preaching, Mr. H. and I will keep the people awake."—*Amer. Jour. of Insanity.*

